# Generation of Functional Insulin-Producing Cells from Neonatal Porcine Liver-Derived Cells by PDX1/VP16, BETA2/NeuroD and MafA

**DOI:** 10.1371/journal.pone.0079076

**Published:** 2013-11-15

**Authors:** Dong-Sik Ham, Juyoung Shin, Ji-Won Kim, Heon-Seok Park, Jae-Hyoung Cho, Kun-Ho Yoon

**Affiliations:** 1 Department of Endocrinology and Metabolism, College of Medicine, The Catholic University of Korea, Seoul, Korea; 2 Seoul St. Mary’s Hospital Convergent Research Consortium for Immunologic Disease, Seoul, Korea; University of Bremen, Germany

## Abstract

Surrogate β-cells derived from stem cells are needed to cure type 1 diabetes, and neonatal liver cells may be an attractive alternative to stem cells for the generation of β-cells. In this study, we attempted to generate insulin-producing cells from neonatal porcine liver-derived cells using adenoviruses carrying three genes: pancreatic and duodenal homeobox factor1 (PDX1)/VP16, BETA2/NeuroD and v-maf musculo aponeurotic fibrosarcoma oncogene homolog A (MafA), which are all known to play critical roles in pancreatic development. Isolated neonatal porcine liver-derived cells were sequentially transduced with triple adenoviruses and grown in induction medium containing a high concentration of glucose, epidermal growth factors, nicotinamide and a low concentration of serum following the induction of aggregation for further maturation. We noted that the cells displayed a number of molecular characteristics of pancreatic β-cells, including expressing several transcription factors necessary for β-cell development and function. In addition, these cells synthesized and physiologically secreted insulin. Transplanting these differentiated cells into streptozotocin-induced immunodeficient diabetic mice led to the reversal of hyperglycemia, and more than 18% of the cells in the grafts expressed insulin at 6 weeks after transplantation. These data suggested that neonatal porcine liver-derived cells can be differentiated into functional insulin-producing cells under the culture conditions presented in this report and indicated that neonatal porcine liver-derived cells (NPLCs) might be useful as a potential source of cells for β-cell replacement therapy in efforts to cure type I diabetes.

## Introduction

Pancreatic islet cell transplantation has proven effective in achieving insulin-independent persistent normoglycemia in patients with diabetes since the Edmonton protocol was reported by Shapiro [1]. This significant progress in diabetes treatment is limited by the shortage of donor organs and the need to follow a lifelong immunosuppressive regimen [2]. Therefore, it is accepted that islet cell transplantation will become widely available only when new sources of islets or pancreatic β-cells are found.

Reprogramming non-endocrine precursors or stem cells into β-cells is considered an alternative option for restoring physiological β-cell mass. The concept of generating insulin-producing cells was inspired in part by studies demonstrating that adult bone marrow cells [3], umbilical cord stromal mesenchymal stem cells [4], limbal stem cells [5], liver cells [6], [7], and pancreatic ductal cells [8] can induce insulin-producing cells via genetic engineering or treatment under various culture conditions.

Pancreatic endocrine differentiation is induced by the sequential expression of specific transcription factors (TFs) during development [9], [10]. Among the many TFs involved in pancreatic development, pancreatic and duodenal homeobox 1 (PDX1) plays a key role in initiating pancreatic organogenesis and maintaining the function of mature β-cells [11]. PDX1/VP16 is a hyperactive version of PDX1 generated by fusing mouse PDX1 to the VP16 activation domain [12]. Many researchers have reported a critical role of other transcription factors, including BETA2/NeuroD [13]–[15] and MafA [16], [17], in β-cell differentiation. Therefore, the combination of these three TFs is considered a useful tool for the transdifferentiation of non-β-cells [18].

Unlike the pancreas, the liver can regenerate efficiently through the proliferation of mature hepatocytes [19]. In addition, the liver and pancreas are developmentally related because both are derived from appendages of the upper primitive foregut endoderm. These two tissues have many characteristics in common, including showing responsiveness to glucose, and both tissues express a large group of specific TFs [20], [21]. Developmental redirection is most likely to occur between tissues that are developmentally related, such as the liver and pancreas [22]. Thus, liver tissue is considered an excellent candidate for generating pancreatic β-cell surrogates.

Most approaches involving hepatocytes have demonstrated that the ectopic expression of TFs converts hepatocytes into insulin-producing cells characterized by stage-specific TF expression in the absence of further differentiation [6], [7], [23]–[27]. Unfortunately, these cells cannot respond to *in vitro* or *in vivo*, which has raised questions about their capacity to produce and secrete insulin as well as to efficiently differentiate into insulin-producing cells. To overcome these obstacles, a new source of cells as well as a combination of pancreatic TFs and the development of a maturation protocol is needed.

In this work, we aimed to overcome current limitations in converting hepatocytes into functional insulin-producing cells by utilizing the neonatal porcine liver as a potential source for generating insulin-producing β-cell surrogates. Neonatal porcine islets have been shown to exhibit many advantages over adult islet cells, including showing an enhanced reproducibility of isolation success and higher yields. In addition, cells from neonatal tissue display a higher potential for proliferation and differentiation than those from adult tissue [4], [28]–[30]. Recently, Leda and colleagues reported that neonatal liver cells express an immature hepatic marker or stem cell marker, and they suggested that small hepatocytes from neonatal livers could be induced to become insulin-producing cells via treatment with high glucose and exendin-4 [25]. These results suggest that the proposed cell/tissue model may offer several advantages as a candidate for substitution cell therapy in diabetes. However, this model is restricted to *in vitro* experiments, without allowing the assessment of *in vivo* cell properties, including the physiological capacity of cells to normalize hyperglycemia in diabetic animal models. Here, we describe the molecular changes and physiological functions in neonatal porcine liver-derived cells *in vitro* following transduction with Ad-PDX1/VP16, BETA2/NeuroD and MafA as well as the optimal culture conditions (high glucose, soluble factors and suspension culture). To this end, we established a stepwise protocol consisting of three steps that is capable of overcoming the limitations of previous protocols for achieving differentiation and maturation. Following our approach using neonatal porcine liver-derived cells, we were able to obtain functional insulin-producing cells capable of lowering hyperglycemia in diabetic animals.

## Results

### Immunohistochemical Analysis of Adult and Neonatal Porcine Liver Sections

**Figure 1:**
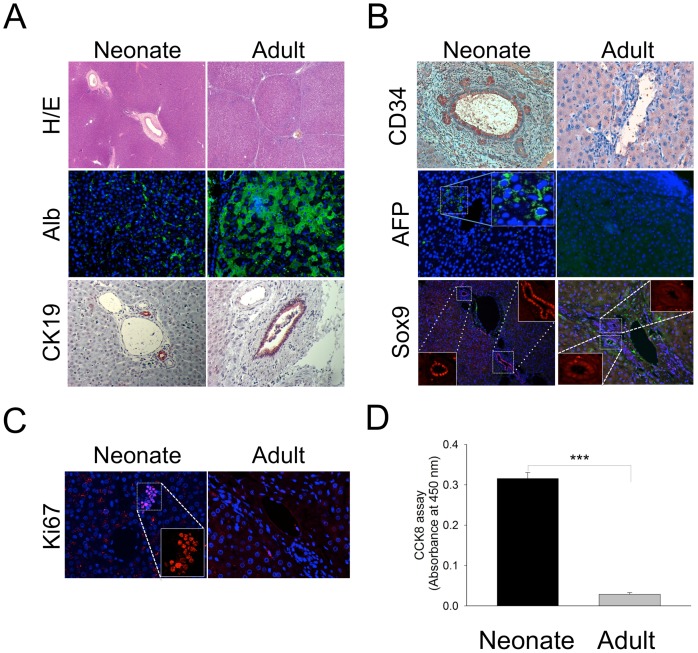
Histological and cellular morphologies in the neonatal and adult porcine liver. (A) Hematoxylin and eosin staining revealed differences in the lobular structures of these 2 tissues (upper panel). Albumin, a marker of mature hepatic cells, was weakly expressed in the neonatal liver compared to the adult liver (middle panel). Cytokeratin 19 (CK19), an epithelial or ductal cell marker, was detected in the liver sections. (B) Immunohistochemical staining to detect CD34 (upper panel), alpha-fetoprotein (AFP; a stem cell marker; middle panel), and sox9 (a marker of early cells in the bile duct; bottom panel) in liver sections. Note that CD34, AFP, and sox9 were only expressed in neonatal tissue and not in adult tissue. (C) Proliferating cells were only detected in neonatal livers via Ki67 staining. (D) CCK-8 activity was measured in primary isolated and cultured liver cells from neonatal and adult livers. The measurements were conducted in three independent experiments. ***P<0.005. Error bar represents the SE.

Hematoxylin/eosin (H/E) staining was performed to compare the characteristics of adult and neonatal livers (Figure 1A). Albumin was strongly expressed throughout the entire adult porcine liver, but its expression was disrupted in the neonatal liver. Moreover, we observed structural units known as liver lobules in adult tissue, but not in neonatal tissue. We also investigated the expression of CD34 (a marker of ductal cells), alpha-fetoprotein (AFP; a marker of immature hepatic cells), and Sox9 (a marker of liver progenitor cells) (Figure 1B). The results of the immunohistochemical analysis indicated that the neonatal porcine liver cells expressed both AFP and CD34, which are markers of immature hepatocytes. However, cells from adult porcine liver rarely expressed both AFP and CD34. The expression level of CK19 did not differ between the 2 groups. In addition, we examined the expression of Ki67, a nuclear marker of proliferating cells, in neonatal and adult livers (Figure 1C). Ki67+ cells were detected in neonatal liver tissue but were not detected in adult liver tissue. The CCK-8 assay was then performed 3 days after isolation, and the results indicated that NPLCs included a greater number of viable cells than adult hepatocytes under normal growth conditions (Figure 1D).

### Ectopic Expression of Adenoviral Vectors in Neonatal Porcine Liver-derived Cells

**Figure 2:**
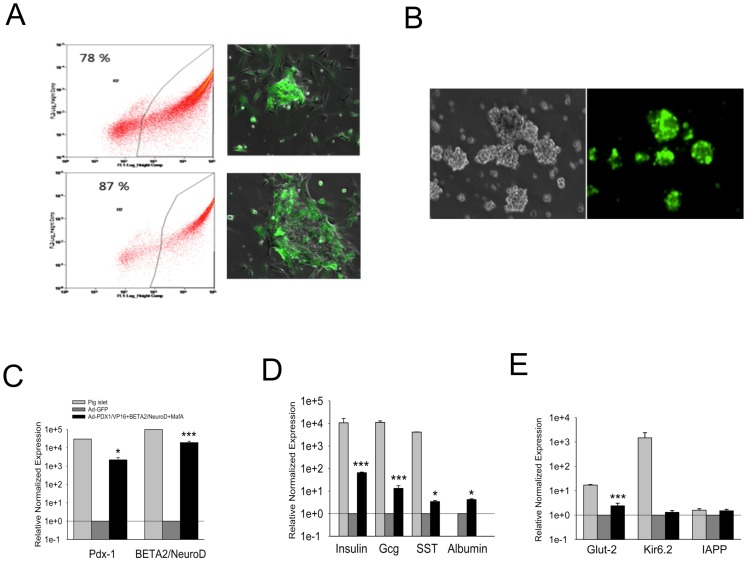
Viral transduction efficiency determined via FACS and relative gene expression determined via quantitative real-time PCR. (A) Various viral titers were tested using green fluorescent protein (GFP) as a reporter gene. Sampling was performed at 72 h after the transduction of Ad-GFP. The obtained transduction efficiency was 78% using a multiplicity of infection (MOI) of 50 after 16 h (upper) and 87% at an MOI of 100 (bottom). (B) Monolayer cells spontaneously formed clusters on low-attachment culture dishes at 24 h after transduction. Most of the clusters expressed GFP well at 72 h after transduction (C–E) qRT-PCR analysis in transduced cells. Sampling was performed during final step of differentiation. Ct values were normalized to GAPDH gene expression within the same cDNA sample. The results are presented as the fold increase based on GAPDH gene expression. All primers were designed based on the pig-aligned sequence from NCBI. The presenteddata are the means of six independent experiments. The meansand standard error values are presented. *P<0.05; **P<0.01; ***P<0.005;Error bars, S.E.

We examined the transduction efficiency obtained using an adenoviral vector expressing green fluorescence protein (GFP). Flowcytometry was employed to determine the efficiency of transduction in NPLCs transduced at an MOI of 50 or 100 for 24 h. At an MOI of 50, we observed an infection rate of 78% in cultured NPLCs after 24 h (Figure 2A, upper). At an MOI of 100, we observed that 87% of cells were infected with Ad-GFP (Figure 2A, bottom). Thereafter, we transferred the infected cells to low-attachment dishes to form islet-like clusters, and we observed the formation of clusters in suspension culture. Interestingly, the cells still expressed GFP at 72 h after infection and exhibited clustering (Figure 2B). These data indicated that genes can be efficiently delivered to NPLCs through adenoviral transduction, without any significant apoptotic effects on the cells. After determining the transduction efficiency, we used a higher titer (100 MOI for 24 h) for the remaining experiments.

### Quantitative Reverse Transcriptase-polymerase Chain Reaction (qRT-PCR)

We performed qRT-PCR to assess the mRNA expression levels of liver- and pancreas-specific transcription factors following cell differentiation (Figure 2C–2E). The results consistently revealed higher expression levels of endogenous PDX1 and BETA2/NeuroD mRNAs in treated NPLCs than in control cells (Figure 2C). These cells expressed numerous pancreatic endocrine markers, including insulin, glucagon (Gcg), somatostatin (SST) and the hepatic marker albumin (Figure 2D). In addition, we observed that the expression of the Glut2 gene was significantly higher in treated NPLCs. However, we did not detect expression of the genes encoding the potassium channel KCNJ11 (Kir 6.2) or islet amyloid polypeptide (IAPP) in either treated or untreated NPLCs (Figure 2E). These data indicated a change in the characteristic liver phenotype of the NPLCs to a β-cell-like phenotype at the molecular level, especially with regard to the expression of PDX-1, BETA2/NeuroD, insulin, and Glut2.

### Phenotype of Treated NPLCs

**Figure 3:**
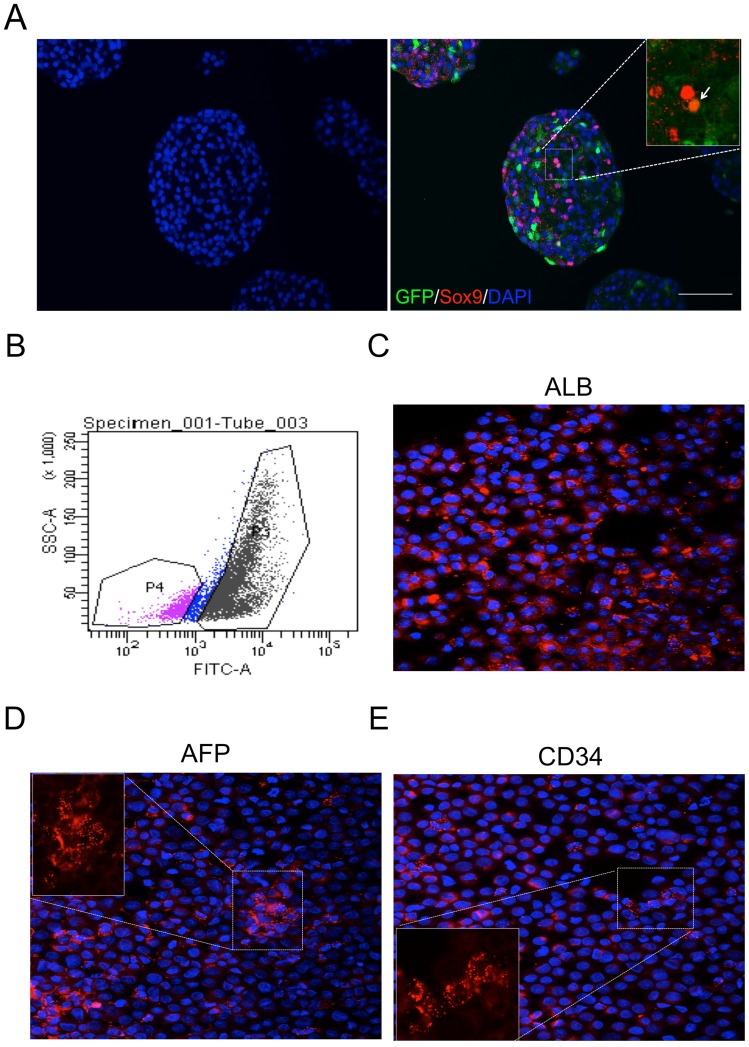
Immunofluorescence staining of NPLCs following the transduction of Ad-GFP. NPLCs were transduced with Ad-GFP 1 day after isolation for 24 h. (A) Sox+ expression in DAPI+ nuclei. Numerous Sox9+ cells were observed in aggregates following (B) FACS sorting of NPLCs. Sampling for FACS was performed at 48 h after transduction. A total of 78% of the GFP+ cells were sorted. (C) Immunofluorescence staining to detect albumin (ALB), alpha fetoprotein (AFP) and CD34.

We investigated Sox9 expression in adenovirus-modified cells (Day 9, see Figure S1) via immunocytochemistry. Numerous Sox9+ cells were detected in the DAPI+ nuclei of adenovirus-modified cell clusters, while GFP+/Sox9+ cells were rarely detected (Figure 3A). In addition, the GFP+ cells expressed markers of immature cells, including AFP and CD34 (Figure 3C–3E). Based on these data, we speculated that GFP+ cells expressing AFP and CD34 might potentially differentiate into cells of other lineages, such as insulin+ cells.

### Glucose Responsiveness of Treated NPLCs Following Trans-differentiation

**Figure 4:**
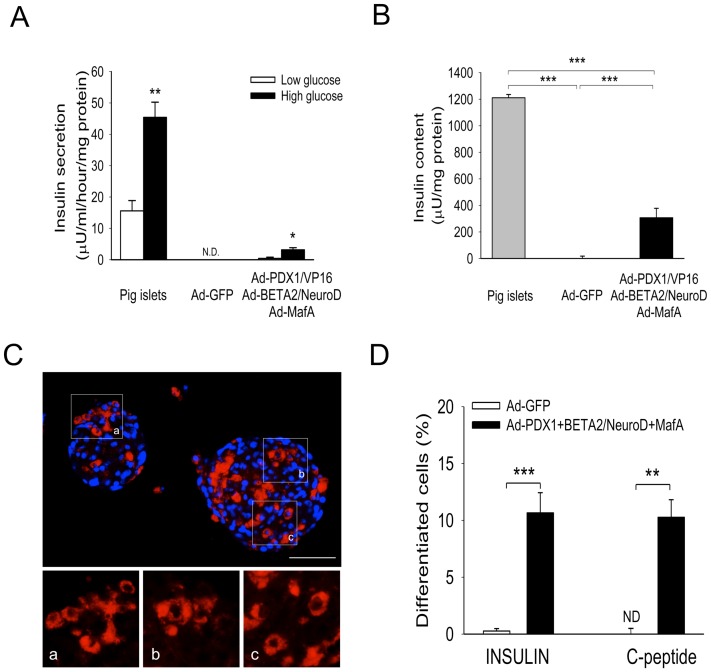
*In vitro* functional analyses and quantification of cellular differentiation. (A) Measurement of glucose-stimulated insulin secretion from NPLCs transduced with Ad-GFP or Ad-PDX1/VP16, BETA2/NeuroD and MafA (n = 3). Islets from adult SNU pigs were used as a positive control. (B) Cellular insulin content. The insulin content was normalized to the amount of total cellular protein (mg). (C) Immunofluorescence visualization of treated NPLCs. Sampling was performed at 6 days after reaggregation, which is the final step of the induction of differentiation. Nuclei are stained with DAPI (a–c) in high-magnification images. (D) Quantification data indicate that the treated NPLCs were trans-differentiated into insulin-producing cells at an efficiency of 11%. Scale bars, 100 µm. *P<0.05;**P<0.01; ***P<0.005; Error bars, S.E.

We examined the glucose responsiveness and cellular insulin content of treated NPLCs (Figure 4A and 4B). Insulin secretion and cellular insulin contents were detectable in treated NPLCs. The amounts of insulin secreted from the treated cells were 0.43±0.1 µUml^−1^h^−1^ per milligram of total cellular protein (1 mM glucose) and 3.2±1.6 µUml^−1^h^−1^ per milligram of total cellular protein (25 mM glucose). The amounts of insulin secreted from adult porcine islets were 15.6±3.3 µUml^−1^h^−1^ per milligram of total cellular protein (2.8 mM glucose) and 45.4±4.9 µU ml^−1^h^−1^ per milligram of total cellular protein (16.8 mM glucose) (Figure 4A). These insulin levels were approximately 7% of those found in normal islets from the pig pancreases. In addition, the cellular insulin content increased to 307.1±71.01 µU ml^−1^h^−1^ per milligram of cellular protein in the treated NPLCs compared to normal rat islets, showing a cellular insulin content of 1,211.03±60.4 µU ml^−1^h^−1^ per milligram of cellular protein (Figure 4B). Overall, these data indicated that the treated NPLCs developed a β-cell-like phenotype at both physiological and molecular levels *in vitro*.

### Immunocytochemical Analysis of Pancreatic Hormones *in vitro*

We investigated the localization of insulin in treated and untreated NPLCs. Insulin was expressed in the cytoplasm of treated NPLCs, but not in the cytoplasm of untreated NPLCs (Figure 4C). We quantified the immunocytochemical data from 3 independent experiments and reported the results as the percentage of total cells. Quantitative analyses showed that 11% of all cells stained positive for insulin (Figure 4D).

### Reversal of Hyperglycemia in STZ-induced Type 1 Diabetic Animals via Transplantation of Transdifferentiated NPLCs

**Figure 5:**
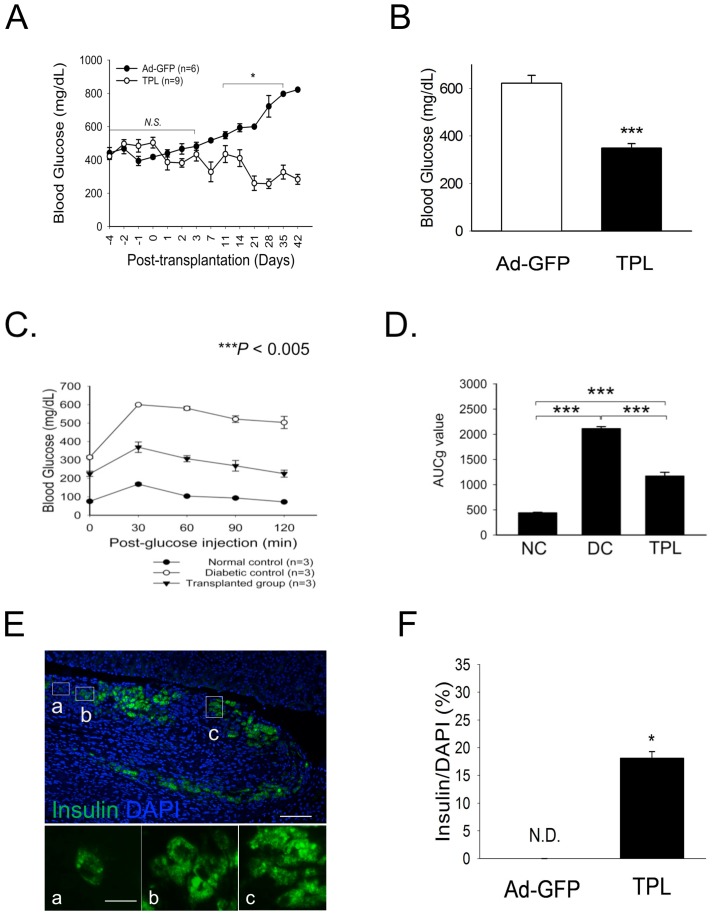
Effect of the subcapsular transplantation of treated NPLCs on hyperglycemia in STZ-induced nude mice. (A) Non-fasting blood glucose levels were monitored for 6 weeks after transplantation. (B) The average blood glucose levels over the recording period, from 7–42 days after transplantation, are reported (Ad-GFP, animals receiving cells transduced with Ad-GFP, n = 6; TPL, animals receiving cells transduced with triple adenoviruses, n = 9). (C) At 6 weeks after transplantation, the intra-peritoneal glucose tolerance test (IP-GTT) was performed in 3 groups: normal control mice (n = 3), STZ diabetic immune-deficient mice (diabetic control, n = 3), and mice receiving cells containing Ad-PDX1/VP16, BETA2/NeuroD, and MafA (transplanted group; TPL, n = 3). (D) The values for the area under the curve for glucose (AUCg) indicate an improvement of diabetes upon transplantation of treated NPLCs (NC, normal control; DC, diabetic control; TPL, transplanted group). The Bonferroni method was used for statistical analysis. (E) At 6 weeks after transplantation, the grafts were harvested. Note the insulin-positive cells in the graft. Nuclei were stained with DAPI. (a–c) High-magnification images.(F) Quantification of insulin-positive cells in the graft. Scale bars, 50 µm. *P<0.05; ***P<0.005; Error bars, S.E.

A schematic timeline of the *in vivo* transplantation analysis is presented in Figure S2A. To evaluate the capacity of treated NPLCs to correct hyperglycemia, we prepared diabetic nude mice by injecting STZ and transplanted treated NPLCs (referred to as the *TPL group*; 10,000 Eq/mouse; n = 9) into the subcapsular space of the kidney. As a control, STZ-induced diabetic nude mice were subjected to transplantation of untreated NPLCs (referred to as the *Ad-GFP group*; n = 6), and 3 diabetic animals received no cells (referred to as the *diabetic control* group). At 7 days before the IP-GTT assays, diabetic control mice were prepared due to the low survival rate of the Ad-GFP group. As noted in Fig. 5A, transplantation of treated NPLCs into diabetic mice resulted in a partial rescue of diabetes. In contrast, the blood glucose levels of the Ad-GFP mice remained elevated (*P*<0.05). We monitored 9 mice in the TPL group and 6 mice in the Ad-GFP group. Approximately 56% of these mice became normoglycemic (<300 mg/dl ), and approximately 44% of these mice remained hyperglycemic (>300 mg/dl), as shown in Figure S2B. The average blood glucose levels observed during the monitoring period (7–42 days after transplantation) remained higher in the Ad-GFP group (580±43.2 mg/dl) than the TPL group (362.4±93.4 mg/dl) (Figure 4B). There was a significant difference between the survival rates of these two groups (Ad-GFP vs. TPL), as shown in Figure S3. All of the mice survived over a period of at least 6 weeks (100% survival) in the TPL group, whereas no mice survived in the Ad-GFP group. At 6 weeks after transplantation, we conducted an IP-GTT to confirm the glucose-lowering effect of the treated NPLCs (Figure 5C). The glucose tolerance was significantly improved in the TPL group compared to the diabetic control group. The area under the curve for glucose (AUCg) was calculated based on data from the IP-GTT (Figure 5D). These data indicated that the transplantation of functional β-cell-like cells can attenuate blood glucose and improve glucose tolerance and survival in diabetic nude mice models.

### Immunohistochemical Analyses of Kidney Grafts from NPLC-transplanted Mice

We observed the presence of insulin-positive cells within kidney grafts 6 weeks after transplantation (Fig. 5E). These insulin-positive cells constituted 18±4.05% of the total graft volume (Figure 5F).

## Discussion

This study clearly demonstrated that neonatal porcine liver-derived cells (NPLCs) could be transdifferentiated into functional insulin-producing cells under the described culture conditions (removal of dexamethasone, adenoviral transduction of three TFs, and reaggregation).

Neonatal porcine liver tissue was used because it is known to exhibit a higher differentiation and proliferation potential [1], [8] than adult liver tissue. In addition, the expression of markers of immature cells, including CD34, AFP and Sox9, as well as Ki67+ cells were observed in the neonatal livers. The presence of cell populations expressing CD34, AFP, Sox9 and Ki67 indicated that the neonatal hepatocytes displayed a superior ability to differentiate into pancreatic endocrine lineage cells compared to adult hepatocytes. Additionally, immunohistochemical analysis detected more Sox9+ and Ki67+ cells in the neonatal liver than the adult liver, where these cells were not detected (Figs. 1B and 1C), suggesting that various cell types were present in the neonatal liver. However, these results did not prove that Ki67+ or Sox9+ cells in neonatal liver tissue are prone to differentiate into insulin+ cells. Therefore, we investigated whether the adenoviral-modified cells expressed markers of immature cells, including CD34, AFP and Sox9, via immunocytochemistry. As expected, a subset of the GFP+ cells expressed markers of immature cells, including AFP and CD34 (Figure 3B–D). However, GFP+ cells did not co-localize with Sox9+ cells (Figure 3A). Based on these data, we speculated that immature cells among isolated NPLCs might differentiate into cells of other lineages, such as insulin+ cells. However, the characterization of cell populations and the origin of insulin+ cells in the neonatal liver will be the focus of future studies.

Several previous studies have focused on the effects of β-cell transcription factors that were overexpressed in the liver or hepatocytes. Ferber’s group reported that *in vivo* transduction of Ad-PDX1 into the mouse liver results in the production of substantial amounts of insulin [31]. Extensive research has been undertaken to investigate the *in vitro* transdifferentiation of adult liver cells from rodents [3], [23], [24], [32], [33] and humans [25], [34] using virus-mediated gene delivery systems as a potential source of β-cells for transplantation into patients with type 1 diabetes. Although these cells transdifferentiate to express insulin, they are clearly far from true pancreatic β-cells, and major obstacles remain to be overcome. One such obstacle consists of the functional limitations of transdifferentiated cells from the liver, and another is the discrepancy between *in vitro* and *in vivo* results. These reports are summarized in Table S1, and efforts by other groups to generate insulin-producing cells from the liver are noted in this Table.

We have previously demonstrated that primary adult mouse hepatocytes can be induced to become insulin-producing cells using Ad-PDX1/VP16, BETA2/NeuroD and MafA [23]. However, we did not elucidate the limitations regarding the low efficiency and immature function of these transdifferentiated cells in secreting insulin.

To improve upon the previous results, we used neonatal porcine liver-derived cells, rather than adult mouse hepatocytes, and we differentiated the hepatocytes in 3 steps. First, we removed dexamethasone from the medium throughout the culturing period because dexamethasone is typically included in media used for culturing primary hepatocytes to maintain their survival and the expression of genes important for the hepatic phenotype, such as albumin [35], [36]. Moreover, dexamethasone suppresses the expansion and differentiation of neonatal porcine pancreatic cell clusters [37]. Therefore, we hypothesized that the removal of dexamethasone would promote efficient transdifferentiation of liver cells towards another cell type of endodermal origin. Second, we attempted to trigger pancreatic gene expression in liver cells using adenoviruses expressing key pancreatic transcription factors. Based on previous studies [12], [16], [18], [23], [31], [38], we first administered PDX1/VP16 to the NPLCs, followed by the administration of a combination of BETA2/NeuroD and MafA. This combination is more reliable and effective than those used previously, especially when administered in a single vector. Finally, we cultured transduced cells in suspension. Cell clusters formed readily in low-attachment dishes within 3 days and were cultured for an additional 6 days.

Following these treatments, the NPLCs exhibited an islet-like phenotype both *in vitro* and *in vivo* and were able to produce and physiologically secrete insulin. These cells also displayed robust expression of numerous pancreatic endocrine markers, including PDX-1, BETA2/NeuroD, SST, Gcg, Glut2, and insulin.

Unexpectedly, Kir6.2 was not expressed in the transdifferentiated NPLCs. This finding may be related to our experimental protocol, involving chronic high glucose exposure during differentiation. Lower Kir6.2 expression in the differentiated NPLCs may have resulted in the 14-fold lower insulin secretion observed compared to that in adult pig islets. Using immunocytochemistry, we also investigated the differentiation efficiency of the treated NPLCs, which was found to be 11%. Importantly, these transdifferentiated cells showed the ability to secrete insulin in response to an increasing glucose concentration. The actual amount of insulin secreted from these cells was calculated as 3.6±1.6 µU ml^−1^h^−1^per mg protein, which was 5 times higher than was previously observed from transdifferentiated mouse hepatocytes. We then investigated the physiological function of these cells in STZ-induced diabetic nude mice via kidney subcapsular transplantation. Our *in vitro* measurements showed that the glucose-stimulated insulin release from a single NPLC was ≈7% of the release from an adult pig pancreatic islet; based on these data, we estimated that the effects of transplantation of 10,000aggregates of NPLCs would be similar to the transplantation of ≈700 pancreatic islets with respect to insulin replacement. Although the transplanted NPLCs could not cause reversion to completely normal blood glucose levels, these cells significantly lowered the blood glucose levels of diabetic animals after approximately 6 weeks and resulted in improvement of glucose intolerance (Table S2 and Figure 5C and 5D). In addition, there was a significant difference in the survival rates between the two groups (Ad-GFP vs. TPL) (Figure S2). We also observed that 18% of the cells were insulin-producing cells, which indicated a higher efficiency of differentiation *in vivo* than *in vitro*. It is likely that the high-glucose conditions resulting from hyperglycemia facilitated the transdifferentiation of liver cells into β-cell-like cells.

The incomplete reversal resulting from NPLC transplantation was due to the significantly lower levels of insulin produced in response to glucose stimulation compared those produced by pancreatic islets (Figure 4A). Future studies will be required to further optimize culture conditions for reprogramming liver cells into pancreatic-like cells to elucidate the molecular mechanisms associated with liver-to-pancreas transdifferentiation and to identify novel candidate genes triggering this differentiation.

Overall, these results suggest that neonatal porcine liver-derived cells could be a useful alternative source for generating β-cell surrogates for use in clinical applications because of their many advantages, which include their cost effectiveness and high differentiation potential. Importantly, it should be investigated whether this approach can be applied to neonatal human liver cells. Additional studies will be required to further optimize the culture conditions for reprogramming liver cells into pancreatic-like cells to elucidate the molecular mechanisms associated with liver-to-pancreas transdifferentiation and to identify novel candidate genes to trigger this differentiation.

## Materials and Methods

### Animals and Reagents

Male Yorkshire pigs (1–3 days old) were purchased from ExpBio (Seoul, Korea). Adult porcine pancreatic islets or livers from adult Seoul National University (SNU) miniature pigs were kindly provided by Dr. Park of Seoul National University (Seoul, Korea). Immunodeficient nude mice were obtained from Charles River (Orientbio, Seoul, Korea). Collagenase P was purchased from Roche Korea Co. Ltd. (Seoul, Korea). An insulin and C-peptide radioactive immunoassay (RIA) kit was obtained from Linco Research Inc. (MO, USA). An insulin antibody was purchased from Zymed laboratories Inc. (San Francisco, USA). Superscript III for cDNA synthesis was procured from Invitrogen (Carlsbad, CA). Oligonucleotides for real-time PCR analysis were synthesized by Bioneer Inc. (Seoul, Korea). SYBR Green® was purchased from Finnzyme (Espoo, Finland). All materials for cell cultivation were obtained from Gibco BRL (Carlsbad, CA), and growth factors (e.g., epidermal growth factor, EGF; and hepatocyte growth factor, HGF) were procured from Peprotech, Korea Inc. (Seoul, Korea). All other chemicals were purchased from Sigma-Aldrich Inc. All animal studies were conducted under a research protocol approved by the Institutional Animal Care and Use Committee (IACUC) of the Catholic University of Korea, Seoul, Korea.

### Cell Isolation

#### Neonatal liver cells

An entire liver (<40–50 g) was dissected from a neonatal pig aged 3 days and transferred to a biosafety hood in chilled Custodiol®HTK solution (Methapharm, Ontario, Canada). A modified two-step perfusion procedure was performed as previously described [5]. Briefly, a 22-G catheter was inserted in the portal vein to perfuse the liver. Prewarmed HBSS was flushed, and 0.5 mg/ml collagenase P was then infused to digest the liver for 15 min. At the end of digestion, an equal volume of washing buffer (HBSS containing 0.4% bovine serum albumin, BSA) was added, and the obtained tissue pellet was collected via centrifugation at 500 rpm at 4°C. The final pellet was poured through a 500 μm stainless mesh and washed three times, then resuspended in DMEM containing 2% FBS.

#### Adult liver cells

Adult liver tissue (<40–50 g) was obtained from adult SNU pigs provided by Seoul National University (Seoul, Korea) and delivered to our laboratory in chilled Custodiol®HTK solution (Methapharm, Ontario, Canada). The isolation process was as described above.

### Induction of Trans-differentiation

#### Adenoviral transduction

NPLCs were transduced with Ad-PDX1/VP16 (referred to as *treated*) or Ad-GFP (referred to as *untreated*) at a multiplicity of infection (MOI) of 100 for 24 h in serum-free DMEM. Fresh medium was then added to the cells transduced with PDX1/VP16. At 24 h after infection, the treated cells were transduced with Ad-BETA2/NeuroD and Ad-MafA (at 50 MOI each) for 24 h.

#### Formation of islet-like clusters and floating culture

Treated or untreated cells were trypsinized and washed with Dulbecco’s Phosphate buffered saline (D-PBS). These cells were then collected in 50 ml conical tubes, counted, suspended (5×10^6^ cells per group) in culture medium and aliquoted into 15 ml conical tubes at a density of 1×10^6^ cells. The cells were subsequently incubated in a 37°C CO_2_ incubator for 2 h. Following incubation, the cells were seeded into ultra-low attachment culture dishes (Corning, NY 14832, USA) to form islet-like clusters in induction medium consisting of DMEM-F12 supplemented with 20 µg/l EGF, 10 µg/l HGF, 10 mMnicotinamide, 1 mM ascorbic acid 2-phosphate, and 10 µg/l mouse betacellulin (mBTC). At 24 h after culturing in suspension, the cells started to form islet-like clusters. The culture medium was renewed every 2 days. Cells were further cultured for 6 days in induction medium without mBTC. Finally, the entire population of cells in each group, without any sorting, was used for *in vitro* analysis or *in vivo* transplantation. Our experimental plan is presented in Figure S1 and 2A.

### Adenoviral Vectors

Recombinant adenoviruses expressing ratPDX1/VP16, hamster BETA2/NeuroD and mouse MafA were kindly provided by Dr. K. Hideaki (Osaka University, Japan). All vectors were generated using the Ad-Easy system (Clontech). All procedures involving adenoviral vectors were performed as previously described [12]. Briefly, the encoding regions of PDX-1/VP16, BETA2/NeuroD and MafA were cloned into a shuttle vector (pAd-Track-CMV). To achieve homologous recombination, 1.0 *μ*g of a linearized plasmid containing PDX1/VP16, NeuroD or MafA and 0.1 µg of the adenoviral backbone plasmid pAdEasy-1 were introduced into electro-competent *E.coli.* BJ5183 cells via electroporation. The resultant plasmids were retransformed into XL-Gold Ultracompetent Cells (Stratagene, La Jolla, CA). The plasmids were then linearized with *Pac* I and transfected into the adenovirus packaging cell line HEK293 using LipofectAMINE (Invitrogen, Carlsbad, CA). The cell lysate was collected from the HEK293 cells 10 days after transfection and added to fresh HEK293 cells. When most of the cells had been killed by the adenoviral infection and detached, the cell lysate was obtained. This process was repeated 3 times. A control adenovirus expressing green fluorescent protein (GFP) was prepared in the same manner. The adenoviral titers were increased to 1×10^10^ plaque forming units (PFU)/ml using the Adeno-X Virus Purification Kit (Clontech). The viral titers were estimated using the Adeno-X Titer Kit (Clontech).

### Determination of Viable Cell Numbers in Normal Culture Conditions via the CCK-8 Assay

The Cell Count Kit-8 (CCK-8, Dojindo, Japan) was employed to quantitatively evaluate cell viability (ref). Briefly, isolated liver cells (4×10^5^) from neonatal or adult pigs were seeded into 35 mm cell culture dishes (day −2, Figure S1-B). After cell adhesion was verified, the cells were grown in growth medium (day −1 to 0) and then preincubated in serum-free medium for 12 h before performing the CCK-8 assay. Following preincubation, the cells were incubated in CCK-8 working solution (1∶10 diluted in DMEM) for 4 h in a humidified incubator. To measure the resultant absorbance, 100 µl of medium from each dish was transferred to a 96-well plate. Data were obtained using a 450 nm absorbance microplate scanner (Versamax ELISA Microplate reader).

### Flowcytometry

On day 3 after transduction, the cells were washed with D-PBS and dissociated in 0.25% trypsin-EDTA (Invitrogen, Carlsbad, CA) for 5 min (see Figure S1-B). The cells were then collected via centrifugation and resuspended in HBSS containing 1% FBS. Propidium iodide (PI) (Calbiochem-Novabiochem, La Jolla, CA) was added to the cells at a final concentration of 50 µg/ml, and the cells were incubated at 37°C for 30 min in the dark. Normal NPLCs without Ad-GFP were prepared for the assay as a negative control. The samples were analyzed on a FACScan flow cytometer (Becton Dickinson Immunocytometry Systems, BDIS) equipped with a 15 mW air-cooled 488 argon-ion laser. GFP-positive cells were filtered through a 530/30 nm bandpass (BP) filter. Orange emission from PI was filtered through a 585/42 nm BP filter. Electronic compensation was employed among the fluorescence channels to remove residual spectral overlap. The photomultiplier tube voltage and spectral compensation were initially set using cells that were positive only for GFP or PI. The obtained data were analyzed with CELL Quest Pro Software (Becton Dickson) and are representative of at least three independent experiments.

### Quantitative Real-Time PCR (qRT-PCR)

At the end of the experiment (day 9; see Figure S1-B), total cellular mRNA was extracted from cells using the TRIZOL reagent (Invitrogen, Carlsbad, CA). As a control, mRNA was extracted from adult SNU miniature pig pancreas tissue. mRNA (1 *μ*g) was reverse-transcribed with 0.5 *μ*g of oligo-dT primers using the Superscript III system. Each cDNA product, obtained according to the manufacturer’s protocol, was diluted to a concentration of 0.1*μ*g/*μ*l in ultrapure water. Aliquots (0.1 µg) of cDNA were used as a template in 20 µl reaction mixtures including 1×SYBR Mastermix, 10 pM concentrations of the primer pairs, and 0.4 µl of ROX reference dye. The porcine gene-specific primers used for qRT-PCR analysis are listed in Table 1, including the NCBI GenBank Accession number.

**Table 1:** Primer information for quantitative PCR.

Target	NCBI #	Sequence	Product size	Tm	Cycle
PDX1	NM_001141984.1	For– tcccgtggatgaagtctacc	265	56	40
		Rev– ttgtcctcctcctttttcca			
INS	NM_001109772.1	For–gcttcttctacacgcccaag	171	56	40
		Rev– ccagctggtagagggaacag			
ALB	NM_001005208.1	For–agtctgccaagctgctgata	115	56	40
		Rev–agccttgggaaatctctggc			
BETA2	AF_177927.2	For–gagagccccctgactgattg	120	56	40
		Rev–gcccgagaagattgatccgt			
Glucagon	NM_214324.1	For–gatcattcccagctccccag	193	56	40
		Rev–gtgttcatcagccactgcac			
Somatostatin	NM_001009583.1	For–gctctctgaacccaaccaga	101	56	40
		Rev–gaaattcttgcagccagctt			
Glut-2	NM_001097417.1	For–tgaggcaattcaaagcagtg	115	56	40
		Rev– acaaaatttcttgcccaacg			
Kir6.2	EU_655630.1	For–tgtgggcggcaataacatct	201	56	40
		Rev–aagatctcgtcggccaggta			
IAPP	GU_396090.1	For– acttgtgcgactcaacacct	111	56	40
		Rev–tgtgttggatcccaccttgg			
GAPDH	NM_002046	For– gacacgatggtgaaggtcgg	195	56	40
		Rev–ttgactgtgccgtggaactt			

Prior to qRT-PCR analyses, we investigated whether the sequences of the primer sets targeting PDX1 and NeuroD cross-reacted with rodent reference sequences through insilico PCR using genome browsing tools from UCSC Genome Bioinformatics (http://genome.ucsc.edu). We confirmed that the sequences used in these experiments did not match any rodent reference sequences and subsequently conducted qRT-PCR using these primers. The specificity of the amplified product was determined via melting peak analysis. The magnitude of the fluorescence signal was determined using the MiniOpticon™ real-time system (Cat# CFB-3120, BioRad). The data were analyzed using Supports OpticonMonitor™ software, which determines mRNA transcript levels using the threshold cycle (C_T_) method based on the C_T_ measurements obtained during the reaction. The quantified values for each target gene were normalized to the house keeping gene, GAPDH, and the relative quantity was normalized to the template obtained from adult pig islets.

### Glucose-stimulated Insulin Secretion (GSIS) and Cellular Contents

To quantify the amount of insulin secreted in response to glucose stimulation, treated NPLCs were cultured in ITS-free medium for 3 days. Following the ITS-free culture period, 2,500 aggregates were washed 3 times with D-PBS and pre-incubated in Krebs Ringer Buffer (KRB) (115 mM NaCl, 5 mM KCl, 2.5 mM CaCl_2_, 1 mM MgCl_2_, 25 mM NaHCO_3_, 10 mM HEPES, pH7.4, and 1 g/l BSA) for 1 h. After washing the cells 3 times with KRB, they were incubated in 1 ml of prewarmed KRB buffer containing 1 or 25 mM glucose for 24 h at 37°C. The medium was then collected and stored at −70°C until measurements of the amount of secreted insulin were performed. To assess cellular insulin contents, the cells were washed 3 times with D-PBS, sonicated in acid-ethanol buffer, and incubated for 24 h at 4°C with gentle shaking. In parallel with the above procedure, adult porcine islets were prepared for GSIS assays from low glucose (2.8 mM) to high glucose (16.8 mM) conditions. Insulin levels were measured using an anti-porcine insulin RIA kit (Millipore), and the quantity of secreted insulin is presented as microunits per milliliter. The measured values were normalized based on the total protein contents from each group, determined using the Bradford assay.

### Streptozotocin (STZ)-induced Diabetic Animal Model

Diabetes was induced via a single intraperitoneal injection of STZ (180 mg/kg in 0.01 M citrate buffer, pH 4.6). Non-fasting blood glucose levels were determined using glucoX (Arkray Factory, Inc. Japan). After 3–4 days, the BG contents measured in STZ-mice were elevated to hyperglycemic levels (randomly checked 3 times per day; 350–500 mg/dl). The STZ-induced diabetic mice were stabilized in a laboratory environment for one week prior to transplantation. These mice were grouped randomly, and each group was divided into two groups for transplantation of cells transduced with Ad-GFP (referred to as the *Ad-GFP* group; n = 6) or Ad-PDX1/VP16, BETA2/NeuroD and MafA (referred to as the *transplanted group, TPL*; n = 9).

A schematic timeline of the in vivo analysis is provided in Fig. S2A.

### Transplantation of Trans-differentiated NPLCs into Diabetic Mice

Cells were transplanted into the right kidney capsule as 10,000 aggregates with an approximate size of 150*μ*m. Six operations were performed for the Ad-GFP group and nine for the transplanted group (TPL). The animals were maintained on a 37°C hotpad until recovery. Blood glucose levels and body weights were recorded until 6 weeks after transplantation. Normoglycemia following transplantation was defined as 3 consecutive blood glucose levels reading less than 300 mg/dl.

### Intraperitoneal Glucose Tolerance Test (IP-GTT)

At 4 and 6 weeks after transplantation, we conducted IP-GTTs in three groups: the normal control (n = 3), diabetic control (n = 3) and TPL (n = 3) groups. At 7 days before the IP-GTT assay, STZ was injected into the intraperitoneal cavities of nude mice (n = 6), after which the mice were stabilized for 7 days. All mice were bled following overnight fasting to establish basal metabolite concentrations and were then injected intraperitoneally with a volume of a 20% glucose solution calculated to deliver a glucose dose of 2 g/kg body weight. Blood samples were collected at 30, 60, 90, and 120 min after injection. Blood glucose levels were measured immediately after sampling from the tail tip using glucoX (Arkray Factory, Inc. Japan), and the area under the glucose curve (AUCg) was calculated.

### Immunohistochemical Staining

Cells were harvested and fixed in 2% paraformaldehyde (PFA; Sigma) at 4°C overnight. The cells were suspended and embedded in 5% LB agarose at 4°C for 2 h. Agarose gel-embedded cells were processed using a standard paraffin-embedding protocol and sectioned at a 4 µm thickness. Kidney grafts from transplanted animals were harvested at 6 weeks after transplantation. Adult or neonatal liver tissues were obtained from adult SNU miniature pigs (∼ 1 year old) or male Yorkshire pigs (3 days old). All tissues were fixed in 4% PFA at 4°C for 16 h and processed as described above. The following primary antibodies were used: polyclonal guinea pig anti-insulin (1∶200); polyclonal rabbit anti-cytokeratin 19 (1∶100; CK-19); polyclonalgoat anti-albumin (1∶100); monoclonal rabbit anti-alpha-fetoprotein (1∶100; AFP); polyclonal goat anti-CD34 (1∶200); polyclonal rabbit anti-sox9 (1∶200); polyclonal rabbit anti-Ki67 (1∶200). Specimens were incubated overnight at 4°C with the primary antibodies. CD34 and CK19 antibodies were detected with biotin-conjugated immunoglobulin IgG. Albumin and AFP were detected using a FITC-conjugated secondary IgG antibody (1∶200). Ki-67 and sox9 were detected with a rhodamine-conjugated secondary IgG antibody (1∶200). Nuclear staining was performed using hematoxylin or 2-(4-amidinophenyl)-1H-indole-6-carboxamidine (DAPI; Invitrogen). Immunofluorescence was detected via fluorescence microscopy (Carl Zeiss, Germany).

### Statistical Analysis

The results are presented as the means± standard error (SE) of at least three independent experiments. A difference was considered significant if a *P* value of less than 0.05 was obtained using Student’s t-test or through one-way analysis of variance (ANOVA) followed by Bonferroni’s post hoc test when multiple comparisons were performed between groups.

## Supporting Information

Figure S1 **Experimental set-up of the present study.** (A) Stepwise protocol for the induction of insulin-producing cells from neonatal porcine liver-derived cells. The procedures were comprised of three steps: isolation, viral transduction, and cluster formation. The media and chemical factors used in the experiments are summarized. (B) Detailed timeline of the experimental plan. In STEP 1, cells were isolated and cultured for experiments (day-2). In STEP 2, cells were transduced with Ad-GFP (Group 1) or Ad-PDX1/VP16 (Group 2) (day0). On day 1, group 2 was transduced with BETA2/NeuroD and MafA (day 1). In STEP 3, groups 1 and 2 were cultured for six days in low-attachment dishes. (B) Timeline of the in vitro experiments. Cells were isolated (day-2), transduced with adenoviruses (day 0), and aggregated (day 3). Finally, the cells were harvested and prepared for the subsequent experiments (*in vivo* transplantation and *in vitro* assays) (day 9).
(TIF)

Figure S2 **In vivo transplantation.** (A) Time-line of the transplantation experiment. Streptozotocin (180 mg/kg) was injected into the intraperitoneal cavity 7 days before transplantation. In vivo experiments were performed in 3 groups: normal control mice(n = 3), mice receiving untreated NPLCs (Ad-GFP; n = 6), and mice receiving treated NPLCs (TPL; n = 9). Blood glucose levels were monitored during the experimental period. Intraperitoneal glucose tolerance tests (IP-GTTs) were performed in 3 groups: the normal control (n = 3), diabetic control (n = 3), and TPL (n = 3) groups at 28 days and 42 days after transplantation. (B) Blood glucose of individuals in Ad-GFP groups (n = 6) (C) Blood glucose of individuals in TPL (n = 9).
(TIF)

Figure S3 **Survival analysis.** Kaplan–Meier survival curves for control diabetic mice receiving untreated NPLCs (Ad-GFP; n = 6) and diabetic mice receiving treated NPLCs (transplanted group, TPL; n = 9).
(TIF)

Table S1 **Studies on liver to pancreas differentiation using pancreatic transcription factors.** The transcription factors that have been ectopically expressed to induce differentiation to an endocrine cell type in an *in vitro* and an *in vivo* model are summarized (Ad, adenoviral vector; HD-Ad, helper dependent adenoviral vector; LV, lentiviral vector; HC, hepatocyte; LEPC, liver epithelial progenitor cell; Tx, transplantation; P.V., portal vein; K, kidney; ND, not detectable; NA, not available).
(DOCX)

Table S2 **The average of blood glucose after transplantation.** Blood glucose averages between control diabetic mice receiving untreated NPLCs (Ad-GFP; n = 6) and diabetic mice receiving treated NPLCs (transplanted group, TPL; n = 9) represents at four time points; at before transplantation, 2 weeks, 4 weeks and 6 weeks.
(TIF)
