# Supplementary figures and images for: Generation of Functional Insulin-Producing Cells from Neonatal Porcine Liver-Derived Cells by PDX1/VP16, BETA2/NeuroD and MafA
Source: PLoS One. 2013 Nov 15;8(11):e79076. doi: 10.1371/journal.pone.0079076 (PMC3829837; doi:10.1371/journal.pone.0079076)

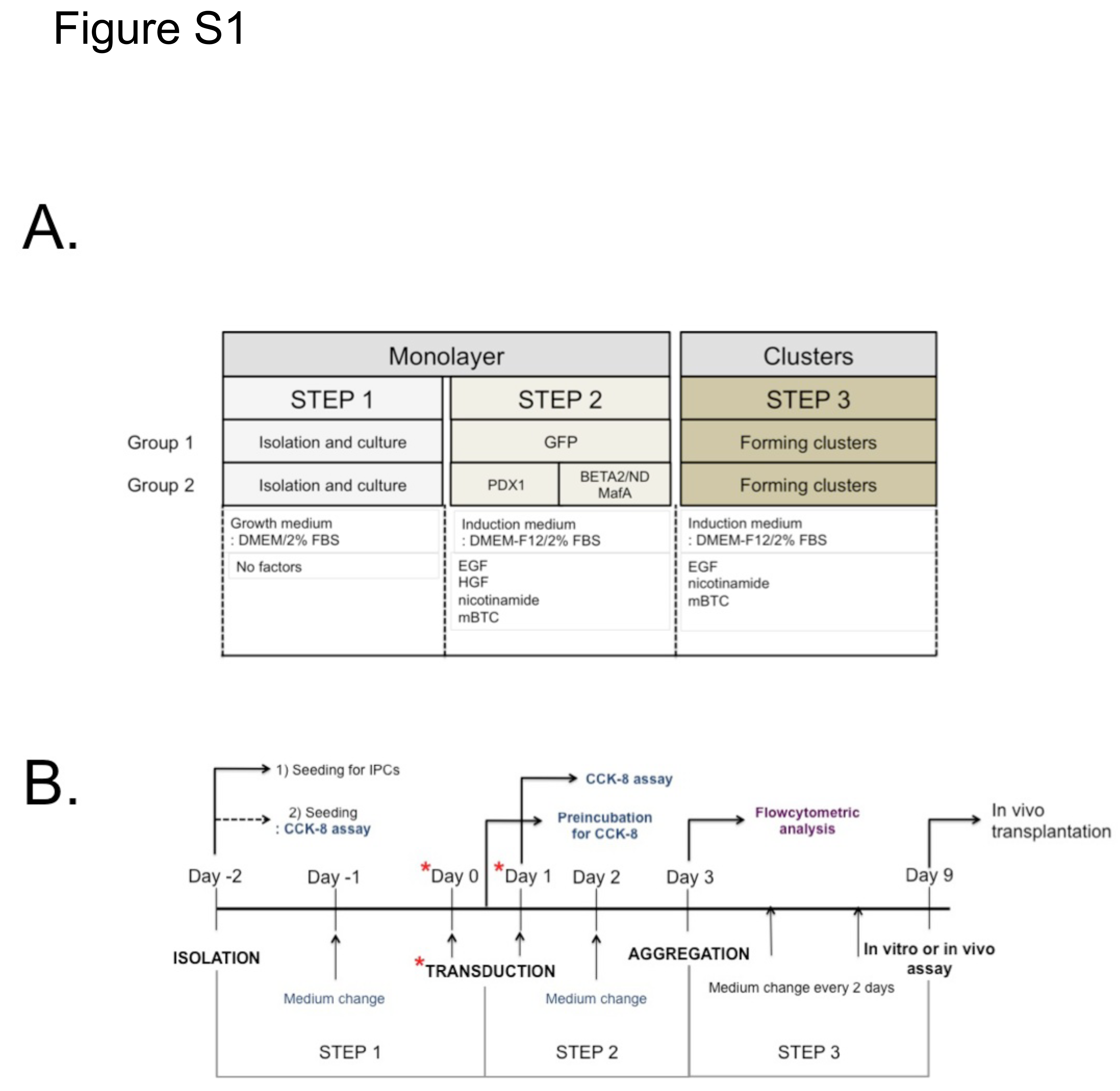

Supplement: Figure S1 — Experimental set-up of the present study. (A) Stepwise protocol for the induction of insulin-producing cells from neonatal porcine liver-derived cells. The procedures were comprised of three steps: isolation, viral transduction, and cluster formation. The media and chemical factors used in the experiments are summarized. (B) Detailed timeline of the experimental plan. In STEP 1, cells were isolated and cultured for experiments (day-2). In STEP 2, cells were transduced with Ad-GFP (Group 1) or Ad-PDX1/VP16 (Group 2) (day0). On day 1, group 2 was transduced with BETA2/NeuroD and MafA (day 1). In STEP 3, groups 1 and 2 were cultured for six days in low-attachment dishes. (B) Timeline of the in vitro experiments. Cells were isolated (day-2), transduced with adenoviruses (day 0), and aggregated (day 3). Finally, the cells were harvested and prepared for the subsequent experiments (in vivo transplantation and in vitro assays) (day 9). (TIF) [file pone.0079076.s001.tif]

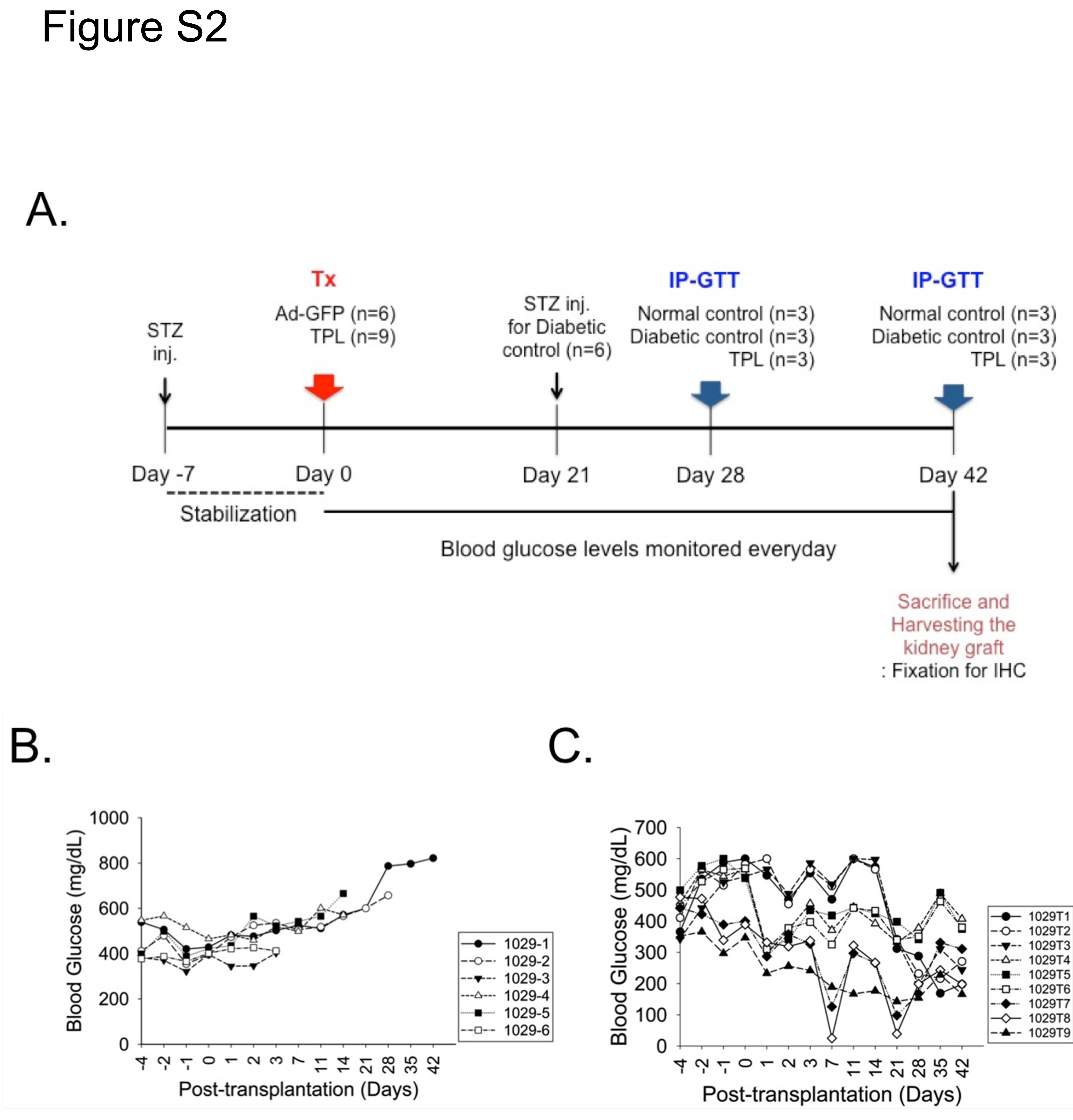

Supplement: Figure S2 — In vivo transplantation. (A) Time-line of the transplantation experiment. Streptozotocin (180 mg/kg) was injected into the intraperitoneal cavity 7 days before transplantation. In vivo experiments were performed in 3 groups: normal control mice(n = 3), mice receiving untreated NPLCs (Ad-GFP; n = 6), and mice receiving treated NPLCs (TPL; n = 9). Blood glucose levels were monitored during the experimental period. Intraperitoneal glucose tolerance tests (IP-GTTs) were performed in 3 groups: the normal control (n = 3), diabetic control (n = 3), and TPL (n = 3) groups at 28 days and 42 days after transplantation. (B) Blood glucose of individuals in Ad-GFP groups (n = 6) (C) Blood glucose of individuals in TPL (n = 9). (TIF) [file pone.0079076.s002.tif]

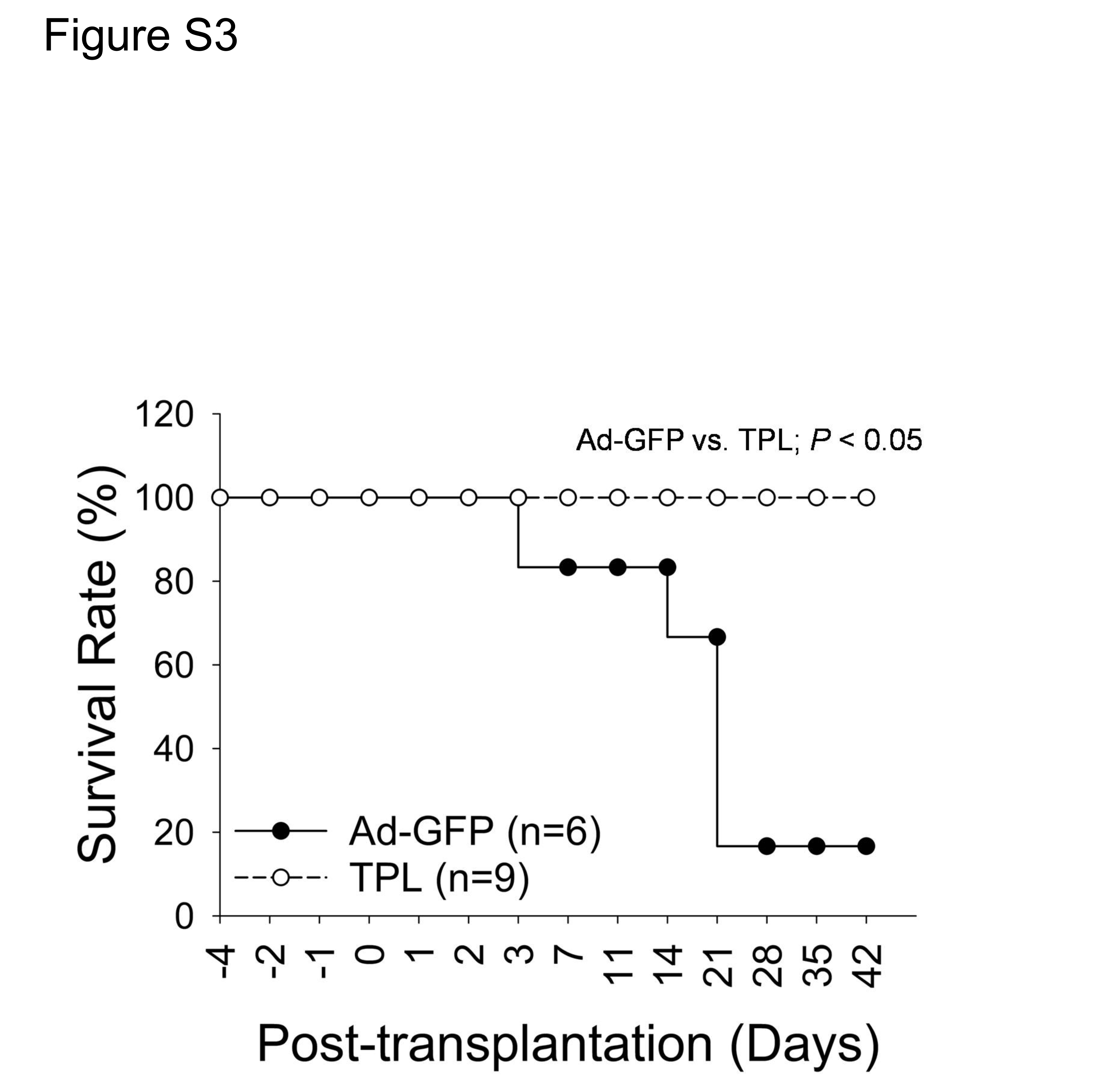

Supplement: Figure S3 — Survival analysis. Kaplan–Meier survival curves for control diabetic mice receiving untreated NPLCs (Ad-GFP; n = 6) and diabetic mice receiving treated NPLCs (transplanted group, TPL; n = 9). (TIF) [file pone.0079076.s003.tif]

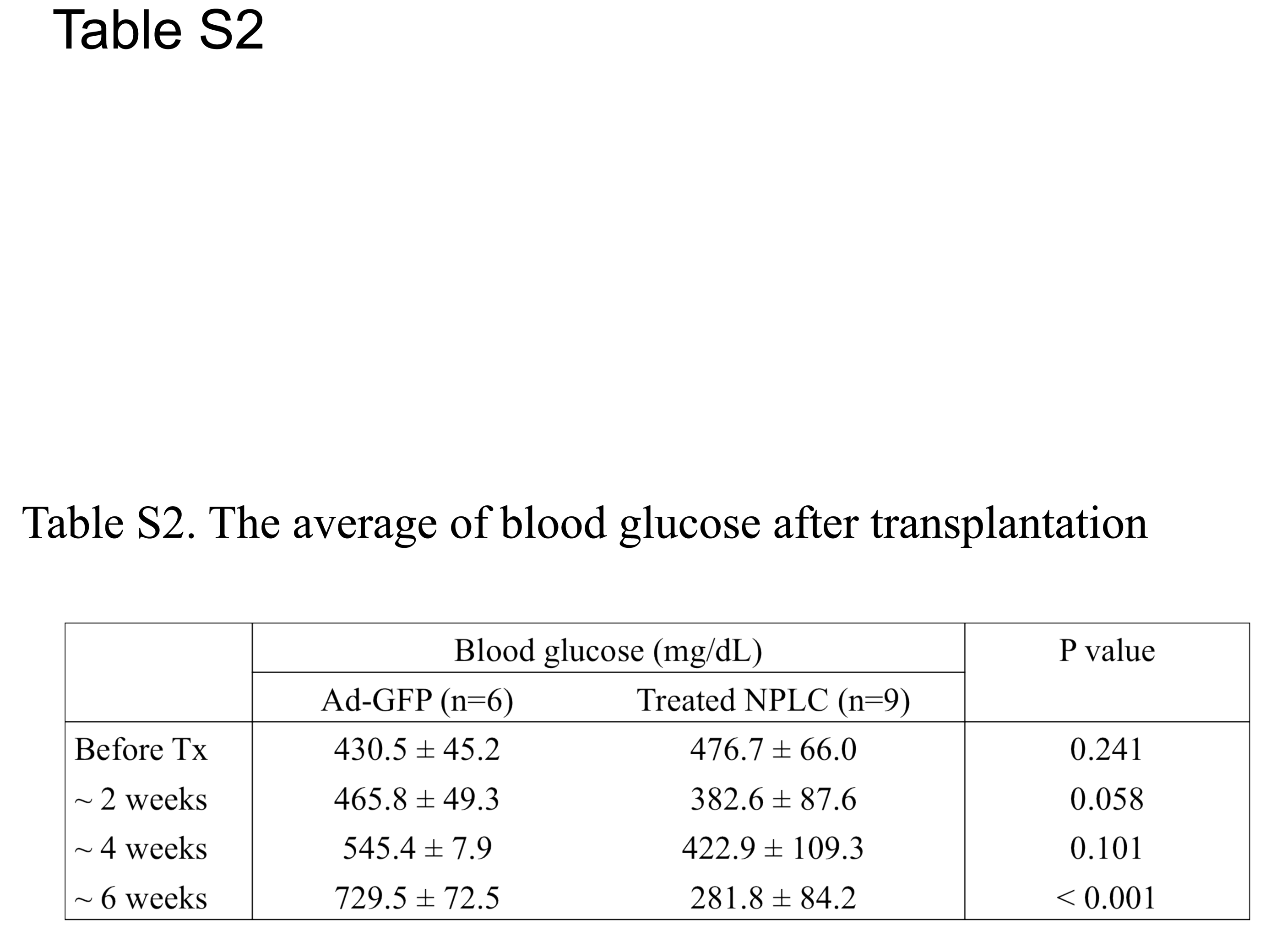

Supplement: Table S2 — The average of blood glucose after transplantation. Blood glucose averages between control diabetic mice receiving untreated NPLCs (Ad-GFP; n = 6) and diabetic mice receiving treated NPLCs (transplanted group, TPL; n = 9) represents at four time points; at before transplantation, 2 weeks, 4 weeks and 6 weeks. (TIF) [file pone.0079076.s005.tif]
