# Supplementary material for: Generation of Functional Insulin-Producing Cells from Neonatal Porcine Liver-Derived Cells by PDX1/VP16, BETA2/NeuroD and MafA
Source: PLoS One. 2013 Nov 15;8(11):e79076. doi: 10.1371/journal.pone.0079076 (PMC3829837; doi:10.1371/journal.pone.0079076)
Supplement: Table S1 — Studies on liver to pancreas differentiation using pancreatic transcription factors. The transcription factors that have been ectopically expressed to induce differentiation to an endocrine cell type in an in vitro and an in vivo model are summarized (Ad, adenoviral vector; HD-Ad, helper dependent adenoviral vector; LV, lentiviral vector; HC, hepatocyte; LEPC, liver epithelial progenitor cell; Tx, transplantation; P.V., portal vein; K, kidney; ND, not detectable; NA, not available). (DOCX) [file pone.0079076.s004.docx]

<Table. S1> Ham et al;

Studies on Liver to pancreas differentiation using Pancreatic transcription factors

| Tissue |  | *In vitro* | | *In vivo* | | |  |
| --- | --- | --- | --- | --- | --- | --- | --- |
| / cells | Viral Vector | Marker of | Secreted | Site | Virus | Days | Reference |
|  |  | ICC | hormones |  | or Cell # |  |  |
| Mouse | Ad-PDX-1 | Insulin | Serum Insulin | P.V. | Viral | 10 d | Ferber et al. (2000) Nat Med |
| Liver |  |  |  |  | Injection |  |  |
|  | Ad- PDX-1 |  |  |  |  |  |  |
| Mouse | PDX-1/VP-16 | Insulin | Data not shown | P.V. | Viral | 14 d | Kaneto et al. |
| Liver | NeuroD |  |  |  | Injection |  | (2005) Diabetes |
|  | Ngn3 |  |  |  |  |  |  |
|  |  |  |  |  |  |  |  |
| Mouse | Ad-MafA | Insulin | Insulin contents | P.V. | Viral | 14 d | Kaneto et al. (2005) |
| Liver | Ad-PDX-1 |  | (~0.15 ng/mg |  | Injection |  | J Biol Chem |
|  | Ad-NeuroD |  | liver) |  |  |  |  |
|  |  |  |  |  |  |  | Kojima et al. (2004) |
| Mouse | HD-Ad-NeuroD | Insulin | Insulin contents | P.V. | Viral | 130 d | Diabetes Res |
| Liver | Betacellulin |  | (~5 pg/mg liver) |  | Injection |  | Clin Pract |
|  |  | Insulin |  |  |  |  |  |
| Human | hTERT with | C-peptide | Insulin contents | K | 2 x 10^6^ | 60 d | Zalzman et al. |
| fetal HC | LV-PDX-1 | NeuroD |  |  |  |  | (2005) Diabetes |
|  |  | Nkx2.2 |  |  |  |  |  |
| Human |  |  |  |  |  |  | Sapir et al. (2005) |
| adult HC | Ad-PDX-1 | Insulin | Insulin | K | 7 x 10^6^ | 60 d | Proc Natl Acad |
| fetal HC |  | PDX-1 | (0.26ng/10^6^ cells) |  |  |  | Sci U S A |
|  |  |  |  |  |  |  |  |
|  | Ad-NeuroD |  |  |  |  |  | Yatoh et al. (2007) |
| Mouse | Ad-Ngn3 | PDX-1 | Data not shown | NA | NA | NA | Diabetes Metab |
| HCs | Ad-PDX-1 |  |  |  |  |  | Res Rev |
|  | Ad-PAX-4 |  |  |  |  |  |  |
|  |  |  |  |  |  |  |  |
| WB-1 | LV-PDX-1 | N.D. | 0.37 ng/mg | K | 2 x 10^6^ | 36 d | Tang et al. (2006) |
|  | LV-PDX-1/VP-16 |  |  |  |  |  | Lab Invest |
|  |  |  |  |  |  |  |  |
| LEPC | Retroviral PDX-1 | C-peptide | 27 ng/10^6^ cells | K | 2 x 10^6^ | 42 d | Jin et al. (2008) |
|  |  |  |  |  |  |  | J Cell Biochem |

Abbreviations: Ad, adenoviral vector; HD-Ad, helper dependent adenoviral vector; LV, lentiviral vector; HC, hepatocyte; LEPC, liver epithelial progenitor cell; Tx, transplantation; P.V., portal vein; K, kidney; ND, not detectable; NA, not available
